# Molecular and functional interactions between AKT and SOX2 in breast carcinoma

**DOI:** 10.18632/oncotarget.6183

**Published:** 2015-10-20

**Authors:** Thorsten Schaefer, Hui Wang, Perihan Mir, Martina Konantz, Tamara C. Pereboom, Anna M. Paczulla, Britta Merz, Tanja Fehm, Sven Perner, Oliver C. Rothfuss, Lothar Kanz, Klaus Schulze-Osthoff, Claudia Lengerke

**Affiliations:** ^1^ Department of Biomedicine, University Hospital Basel, Basel, Switzerland; ^2^ Department of Internal Medicine II, University Hospital Tuebingen, Tuebingen, Germany; ^3^ Interfaculty Institute of Biochemistry, University of Tuebingen, Tuebingen, Germany; ^4^ Women's Hospital, University Hospital Duesseldorf, Duesseldorf, Germany; ^5^ Institute of Pathology, University of Luebeck, Luebeck, Germany; ^6^ German Cancer Consortium (DKTK) and German Cancer Research Center (DKFZ), Heidelberg, Germany; ^7^ Clinic for Hematology, University Hospital Basel, Basel, Switzerland

**Keywords:** SOX2, AKT, breast carcinoma, cancer stem cells, clonogenicity

## Abstract

The transcription factor SOX2 is a key regulator of pluripotency in embryonic stem cells and plays important roles in early organogenesis. Recently, SOX2 expression was documented in various cancers and suggested as a cancer stem cell (CSC) marker. Here we identify the Ser/Thr-kinase AKT as an upstream regulator of SOX2 protein turnover in breast carcinoma (BC). SOX2 and pAKT are co-expressed and co-regulated in breast CSCs and depletion of either reduces clonogenicity. Ectopic SOX2 expression restores clonogenicity and *in vivo* tumorigenicity of AKT-inhibited cells, suggesting that SOX2 acts as a functional downstream AKT target. Mechanistically, we show that AKT physically interacts with the SOX2 protein to modulate its subcellular distribution. AKT kinase inhibition results in enhanced cytoplasmic retention of SOX2, presumably via impaired nuclear import, and in successive cytoplasmic proteasomal degradation of the protein. In line, blockade of either nuclear transport or proteasomal degradation rescues SOX2 expression in AKT-inhibited BC cells. Finally, AKT inhibitors efficiently suppress the growth of SOX2-expressing putative cancer stem cells, whereas conventional chemotherapeutics select for this population. Together, our results suggest the AKT/SOX2 molecular axis as a regulator of BC clonogenicity and AKT inhibitors as promising drugs for the treatment of SOX2-positive BC.

## INTRODUCTION

Pluripotency-associated proteins like SOX2 and OCT4 are key regulators of embryonic stem cells and foster the reprogramming of terminally differentiated somatic cells back to a pluripotent stem cell state [1]. SOX2 is furthermore a major regulator of embryonic development and more recently was demonstrated to determine cellular identity in certain adult stem and progenitor cells [2]. Consistent with the notion that stemness and embryonic pathways can play oncogenic roles, SOX2 expression was documented in several cancers, especially of endodermal, epithelial and neural origin [3–13]. In the breast, SOX2 expression has not been reported in healthy tissues but is detectable across different breast carcinoma (BC) subtypes [14] and particularly prominent also in certain BC-derived metastases [15]. Interestingly, SOX2 expression in BC is mostly confined to a minor subset of tumor cells and detectable at early stages of the disease as well as at relapse, suggesting that it is involved in BC stem cell biology and might represent a genetic driver event [14, 16].

Another major molecular regulator of both embryonic and cancer stem cell self-renewal is the kinase AKT. The canonical PI3K/AKT pathway is known to influence cell metabolism, growth, proliferation and survival and its deregulation is a common determinant in various cancers [17–19]. In healthy mammary epithelial cells, constitutive PI3K/AKT signaling supports the outgrowth of a stem cell population, which can be antagonized by the PI3K/AKT cross-reactive inhibitor perifosine [20]. Furthermore, inhibition of AKT was shown to affect cancer stem cell populations including breast CSCs [21, 22], the underlying molecular details however remain largely unknown.

In the present study we hypothesize that AKT influences BC stem cells by regulating their SOX2 protein levels. We employ the tumor sphere formation assay as a surrogate assay identifying clonogenic tumor cells with CSC-like features in BC cell lines as well as patient-derived cells [23, 24]. We further demonstrate that in BC cells AKT directly interacts with SOX2 and stabilizes the protein by promoting its nuclear localization. Inhibition of AKT kinase activity induces successive proteasomal clearance of SOX2 protein in the cytosol. Underscoring the particular significance of this post-translational regulatory circuit, ectopic overexpression of *SOX2* rescues clonogenicity and *in vivo* tumorigenicity in AKT inhibitor-treated BC cells. Further supporting the notion that disease-initiating breast CSCs are dependent on AKT signaling, treatment with AKT inhibitors suppresses total cell growth, whereas conventional cytostatics impose a selective advantage on BC cells with active *SOX2*-regulatory elements. Therefore, inhibition of the AKT pathway may provide additional benefit for the treatment of SOX2-positive BC patients.

## RESULTS

### The role of *SOX2* in breast CSCs

**Figure 1:**
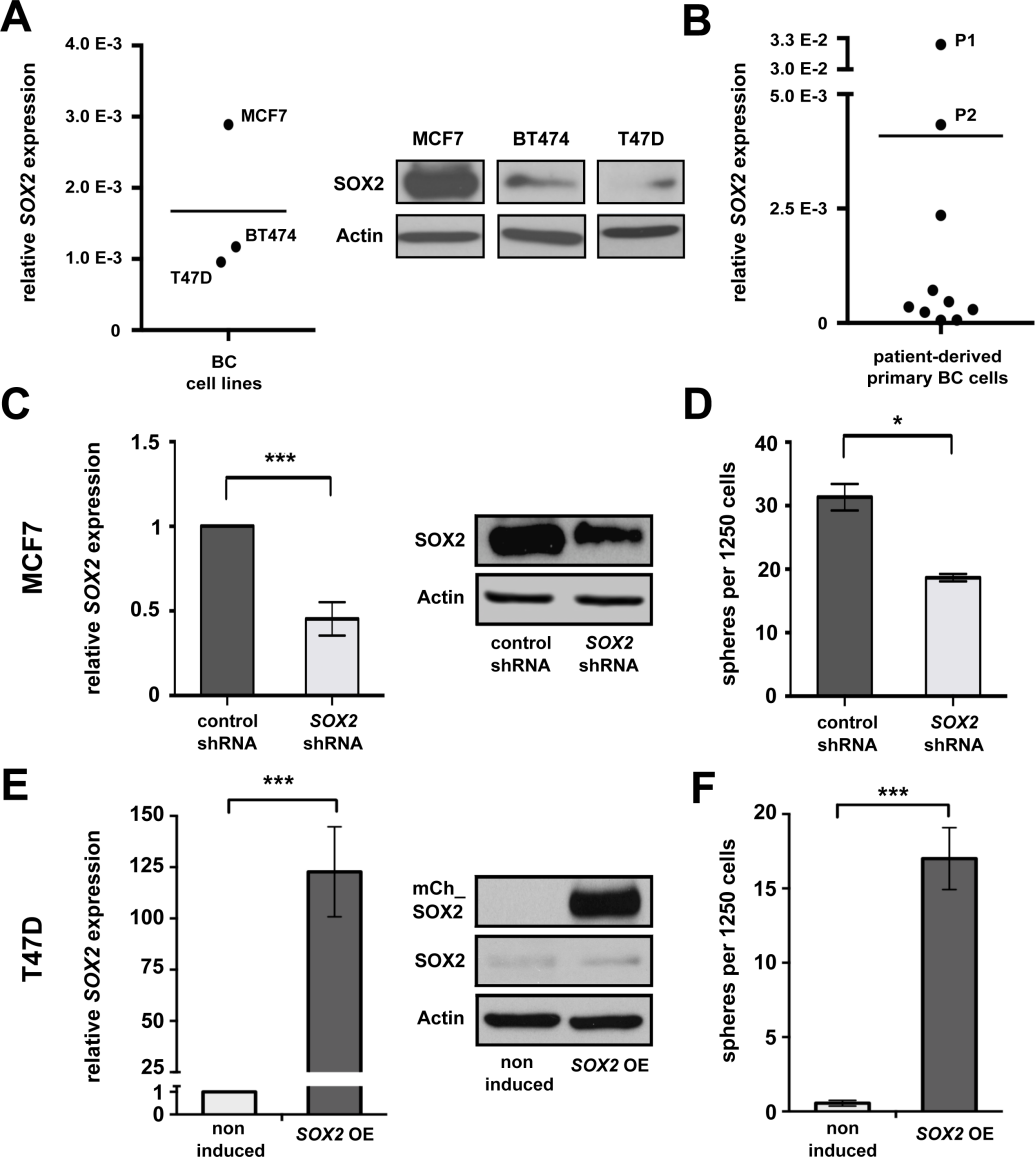
SOX2 is expressed in BC and promotes clonogenicity **(A)** Endogenous SOX2 mRNA (left) and protein (right) expression in BC cell lines MCF7, BT474, and T47D propagated under standard (2D) cultivation conditions. Indicated are mRNA expression levels relative to *GAPDH*. Midline illustrates average *SOX2* expression in the three cell lines analyzed. Actin is shown as a protein loading control. **(B)** Relative *SOX2 mRNA* expression in 10 primary patient-derived BC samples (P1 and P2: samples showing highest endogenous *SOX2* expression, midline to illustrate average). **(C)** Reduced *SOX2* mRNA and protein expression, and **(D)** impaired sphere formation in MCF7 cells transduced with *SOX2* shRNA vs. control lentiviral particles. **(E)** Inducible *mCherry-SOX2* expression in stably transfected T47D cells at 24 hours of induction with 1 μg/ml of doxycycline, as verified by qRT-PCR (left) and immunoblotting (right). **(F)** Ectopic expression of a *mCherry-SOX2* fusion protein (*SOX2 OE*) induces sphere formation in T47D cells. Samples were incubated in 3D medium in the absence or presence of doxycycline (1 μg/ml) for 5 days.

We initially investigated *SOX2* mRNA expression in eight human BC cell lines available in the laboratory (Figure 1A and Supplementary Figure 1). Of these, MCF7, BT474 and T47D cells were selected for further analysis to cover a dynamic range of endogenous SOX2 expression levels (Figure 1A). The remaining cell lines showed modest *SOX2* expression under standard cultivation conditions (2D), but a clear induction of *SOX2* mRNA under 3D conditions that favor the outgrowth of stem cells (Supplementary Figure 1). SOX2 expression was additionally examined on mRNA level in a panel of 10 patient-derived primary cells (Figure 1B). Two SOX2-expressing samples (P1 and P2) were selected for reference experiments.

To verify a functional significance of *SOX2* for BC clonogenicity and to assure its relevance in the particular experimental settings used here, we first investigated the effect of *SOX2* knockdown and inducible overexpression on tumor sphere formation *in vitro*. To this end, MCF7 cells displaying a high endogenous SOX2 expression were treated with two specific *SOX2* shRNAs or respective control GFP-lentiviral particles and correctly transduced cells were isolated by flow cytometry. Effective knockdown of *SOX2* expression in GFP-positive cells was verified by qRT-PCR and immunoblotting (Figure 1C and Supplementary Figure 2). Confirming functional relevance for clonogenicity, *SOX2* knockdown cells displayed a significantly reduced sphere formation capacity in comparison to control cells (Figure 1D, Supplementary Figure 2C, and [25]). To monitor a stimulatory effect of SOX2 on sphere formation, the human *SOX2* gene was N-terminally fused to *mCherry*, cloned under the control of a doxycycline-dependent Tet_ON_ induction system, and lentivirally integrated in T47D cells that showed low endogenous *SOX2* expression (see above). Transduced cells were selected via puromycine resistance and efficient induction of *SOX2* expression following doxycycline treatment confirmed by qRT-PCR and immunoblotting (Figure 1E). Indeed, spheres formation was only observed from SOX2-induced T47D cells, whereas mock-treated control cells were only able to associate in irregularly shaped aggregates (Figure 1F and Supplementary Figure 3).

### AKT inhibition targets clonogenic BC cells

**Figure 2:**
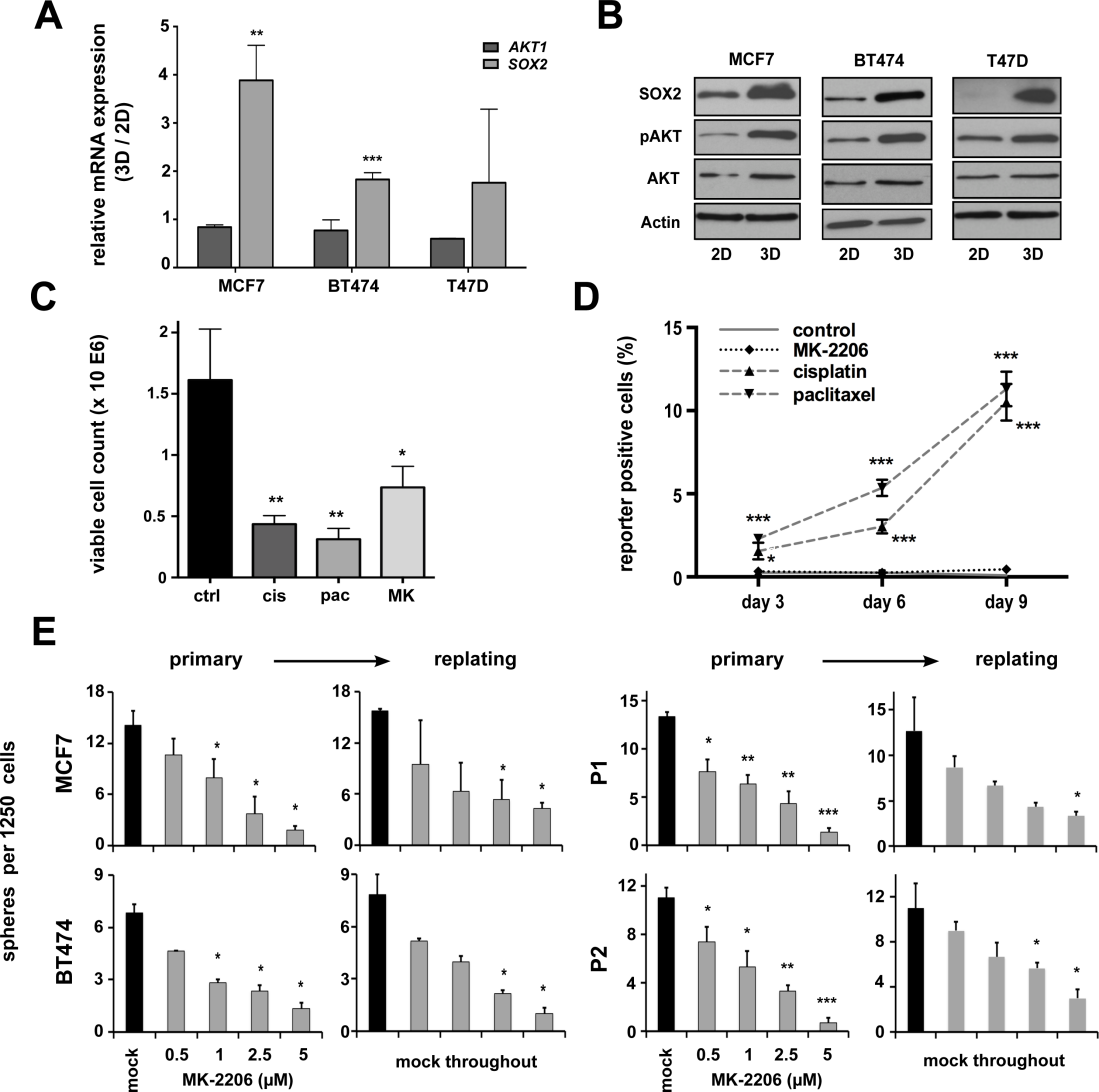
pAKT expression is induced in putative breast CSCs and regulates BC clonogenicity **(A)** Induction of *SOX2* but not *AKT1* gene expression in BC cell lines grown under conditions enriching for CSCs (3D) versus conventional cultures (2D). Indicated are fold changes in expression of the indicated target genes (ΔΔ*C*t of either *AKT1* or *SOX2* mRNA relative to *GAPDH*) in cells grown under 3D versus 2D conditions (3D/2D). **(B)** Corresponding immunoblots document co-induction of SOX2 and pAKT proteins in 3D-cultures, whereas total AKT levels remain largely unchanged. **(C)** Treatment with conventional chemotherapeutic drugs (cisplatin, cis, 5 μM; paclitaxel, pac, 5 nM) or the AKT inhibitor MK-2206 (MK, 1 μM) inhibits the growth of MCF7 cells (50.000 cells seeded, 72 hour follow-up). **(D)** Enrichment of SRR1-expressing putative CSCs in cisplatin or paclitaxel but not MK-2206 treated cells. Indicated is the percentage of SRR reporter-positive MCF7 cells in the surviving cell fraction, as detected by flow cytometry at indicated times. Dead cells were eliminated by DAPI staining and analyses performed on the gated live cell population. **(E)** Dose-dependent suppression of sphere formation by MK-2206 in primary and replating sphere assays (black bars: mock-treated cells; grey bars: MK-2206-treated cells). Note that in replating assays sphere formation was impaired despite the removal of inhibitor. BC cell lines (left), patient-derived primary BC cells (right).

Activating mutations in the AKT pathway are amongst the most frequent somatic aberrations observed in breast cancer [26]. Furthermore, the PI3K/AKT pathway has been implicated in healthy and malignant breast stem cell biology [20]. Supporting these notions, we could show an induction of functionally active pAKT (i.e. AKT carrying a pSer473 auto-phosphorylation signature) along with enhanced SOX2 expression in 3D- versus 2D-cultured cells, albeit total AKT levels remained largely unchanged (Figure 2A and 2B). We therefore reasoned that AKT activity and SOX2 expression could be functionally linked in BC stem cells.

To validate this assumption and to test whether AKT inhibitors may effectively target SOX2-positive breast CSCs, a SRR (*SOX2* regulatory region 1)-based stem cell reporter was stably introduced into the MCF7 cell line [24, 27]. Treatment with conventional cytostatics (e.g. cisplatin, paclitaxel) clearly reduced overall cell growth (Figure 2C), but enhanced the frequency of reporter-positive CSCs in the surviving cell fraction (Figure 2D). By contrast, the pan-AKT inhibitor MK-2206 impaired overall BC cell growth, but did not allow the selective outgrowth of *SOX2*-positive cells (Figure 2C and 2D).

Next, we performed sphere formation assays in presence or absence of MK-2206. Indeed, AKT inhibition resulted in a dose-dependent reduction of sphere formation throughout all analyzed BC cell lines and primary cells (Figure 2E). Taken together, AKT kinase activity influences CSC functions and is a prerequisite for BC cell clonogenicity.

### pAKT is an upstream regulator of SOX2 protein expression in BC

**Figure 3:**
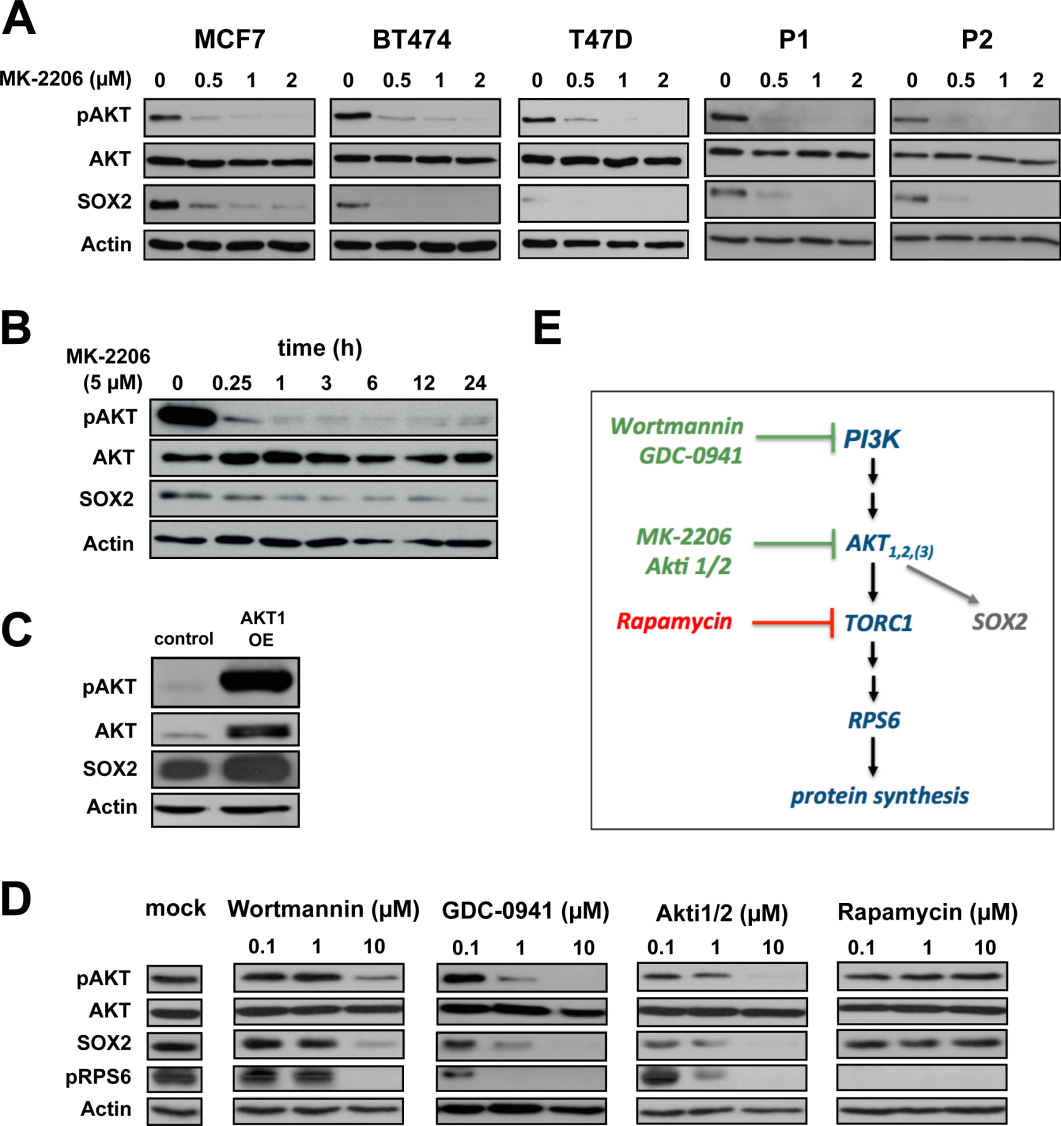
AKT is an upstream regulator of SOX2 protein expression **(A)** Dose-dependent co-depletion of pAKT and SOX2 proteins following MK-2206 treatment in BC cell lines (MCF7, BT474, T47D) and patient-derived cells (P1, P2) within 48 hours of incubation. Note the grossly unaltered levels of total AKT. Anti-actin staining is shown for reference. **(B)** Temporal resolution of pAKT and SOX2 protein expression in MCF7 cells upon AKT inhibition throughout an observation window of 24 hours. **(C)** Transfection with myristoylated *AKT1* induces both pAKT and SOX2 protein expression in MCF7 cells. **(D)** Confirmation of SOX2 protein depletion by the alternative AKT kinase inhibitor Akti1/2 and upstream PI3K inhibitors (wortmannin and GCD-0941) in MCF7 cells. AKT downstream inhibitor rapamycin has no impact on SOX2 expression, instead. Functional integrity of reagents was verified by uniform depletion of pRPS6. Mock-treated control lanes are shown at the left. **(E)** Schematic illustration of the canonical PI3K/AKT/TORC1 pathway. Green: drugs that impair SOX2 protein expression.

Since both SOX2 and pAKT proteins regulate BC clonogenicity, and AKT kinase inhibitors effectively target cells with active *SOX2*-regulatory elements (SRR), we hypothesized that pAKT and SOX2 molecularly interact in breast CSCs. To further explore this notion, SOX2 expression was analyzed in BC cells treated with the pan-AKT inhibitor MK-2206. Indeed, profoundly reduced SOX2 protein levels were observed along with pAKT inhibition upon treatment with MK-2206 (Figure 3A and 3B). This inhibitory effect was dose-dependent, commenced successively, and was consistently observed throughout all analyzed cell lines and patient-derived BC samples. Conversely, induction of pAKT upon transfection with a myristoylated *AKT1* construct clearly up-regulated SOX2 protein (Figure 3C). Together, these data suggest that SOX2 is a pAKT downstream target. To further explore this hypothesis and to control for putative off-target effects of MK-2206, the upstream PI3K inhibitors wortmannin and GDC-0941, as well as the alternative AKT inhibitor Akti1/2 were used to block AKT kinase activity. SOX2 protein depletion was uniformly observed in all these conditions (Figure 3D and 3E), confirming a functional dependence of SOX2 protein expression on canonical PI3K/AKT signaling. Importantly, inhibition of the AKT-downstream target mTOR by rapamycin did not suppress SOX2, albeit efficient inhibition of RPS6 phosphorylation confirmed drug efficacy (Figure 3D). We conclude that AKT kinase is an immediate upstream regulator of SOX2 turnover in BC, and that the disappearance of SOX2 protein in AKT-inhibited cells is not primarily explained by altered *de novo* protein synthesis (Figure 3E).

### SOX2 expression restores clonogenicity and *in vivo* tumor initiation capacity in anti-AKT treated BC cells

**Figure 4:**
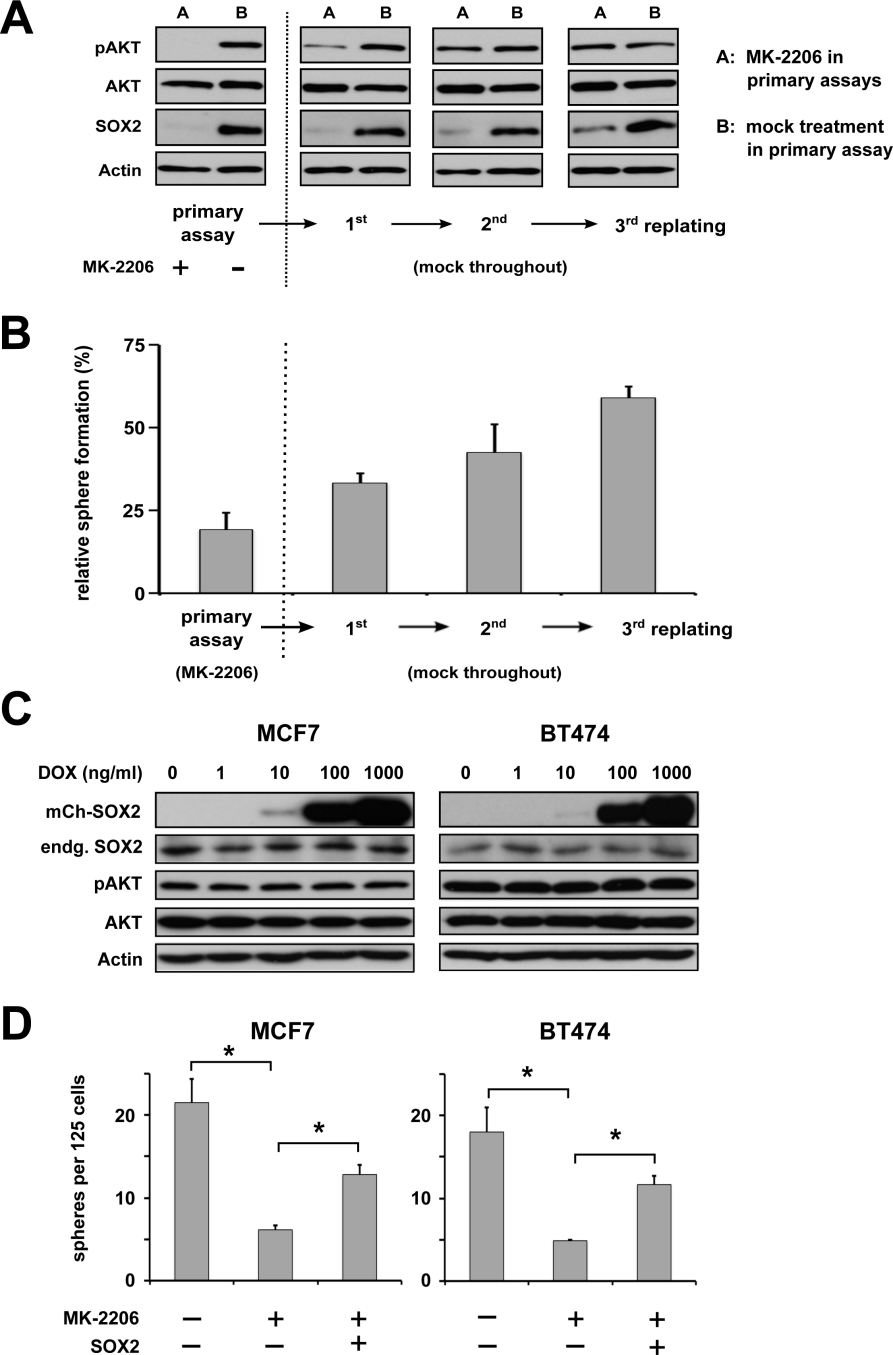
AKT regulates BC clonogenicity via SOX2 **(A)** Primary and serial replating sphere assays document a tight dependence of SOX2 protein expression and **(B)** clonogenicity on pAKT. MK-2206 treatment was ceased after the primary assay cycle. Note a successive recurrence of pAKT and slightly delayed also SOX2 protein in replating assay cycles, which coincides with restoration of sphere formation capacity. Indicated is the percentage of sphere formation relative to replated, mock-treated controls. **(C)** and **(D)** Dose-dependent ectopic induction of a *mCherry-SOX2* fusion protein in the indicated BC cell lines does not affect endogenous SOX2 and pAKT/AKT protein levels (C), but rescues sphere formation in MK-2206-treated cells (D), indicating that SOX2 is as a functional downstream target of pAKT (left: untreated controls, center: 5 μM MK-2206 followed by mock treatment, right: 5 μM MK-2206 and subsequent induction of *mCherry*-*SOX2*).

Interestingly, BC cells treated with MK-2206 showed a dose-dependent reduction in sphere formation not only in primary but also in replating sphere assays where MK-2206 was not anymore added to the cultures (Figure 2E, right panels). To further explore whether this effect was due to continuous pAKT suppression in the absence of the inhibitor or due to effects on cell fate established during the brief treatment window, additional serial replating experiments and corresponding immunoblot analyses were performed. Indeed, while effective suppression of both pAKT and SOX2 protein by MK-2206 was confirmed under 3D cultivation conditions (Figure 4A, left), a sequential re-appearance of pAKT and subsequently also of SOX2 protein was noted upon depletion of the inhibitor. Matching these molecular results, a gradual recovery of sphere formation capacity was observed (Figure 4A and 4B).

To more directly investigate the functional relevance of SOX2 as a downstream pAKT target, SOX2 was ectopically expressed in MK-2206 treated BC cells using a conditional lentiviral *mCherry-SOX2* fusion construct. Efficient dose-dependent induction of *mCherry-SOX2* by doxycycline was confirmed by immunoblot analysis and fluorescence microscopy, and had no overt effect on endogenous AKT/pAKT levels (Figure 4C and Supplementary Figure 4). Supporting the notion that SOX2 is a downstream target of AKT, enforced SOX2 expression partially rescued sphere formation in AKT-inhibited cells (Figure 4D). However, SOX2-expressing spheres derived from AKT inhibitor-treated cells displayed a growth disadvantage in comparison to mock-treated controls suggesting that, in contrast to clonogenicity, proliferation-related defects may not be concomitantly rescued by SOX2. This assumption was supported by cell-cycle analyses, revealing a reduced cell proliferation in AKT-inhibited cells that was not rescued by *SOX2* expression. Furthermore, treatment with AKT inhibitors impaired the expression of several cell cycle-regulators (e.g. cyclin D1, cyclin E, and CDK2), which could not be restored by *SOX2* expression (Supplementary Figure 5).

**Figure 5:**
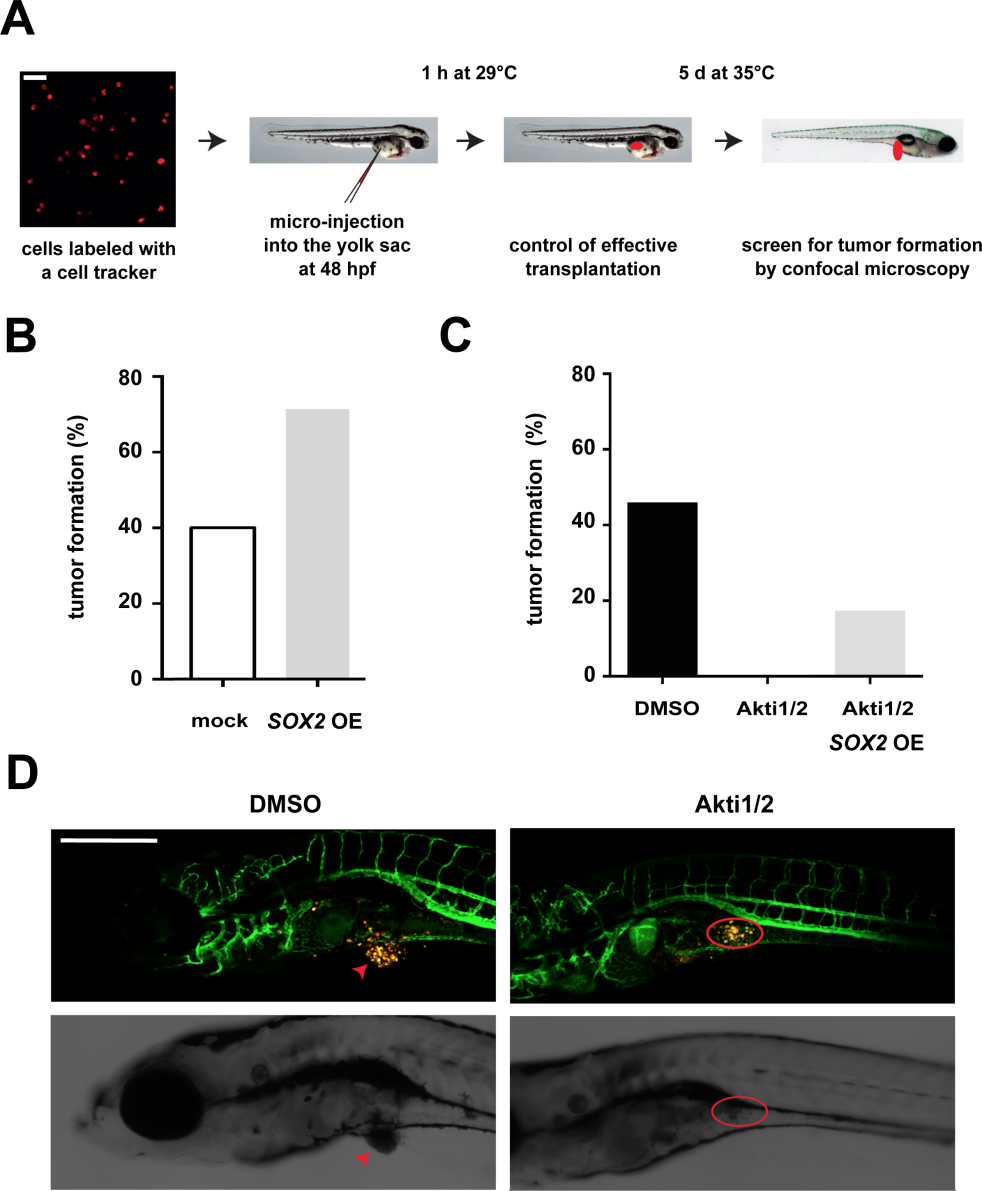
Influence of the AKT/SOX2 axis on *in vivo* tumorigenicity **(A)** Schematic illustration of the zebrafish xenotransplantation procedure and assay. Scale bar: 50 μm. **(B)** *SOX2* overexpression facilitates *in vivo* tumor induction. Shown are percentages of fish with tumors upon transplantation with SOX2-overexpressing versus control T47D cells (75 cells per fish and 10 or more fish for each condition). **(C)** AKT kinase inhibition by Akti1/2 (5 μM) prevents tumor formation in T47D xenotransplanted fish. However, tumor formation in AKT inhibitor-treated embryos is partially restored by concomitant *SOX2* overexpression. At least *n* = 10 embryos were analyzed per group. **(D)** Representative confocal pictures of T47D-induced tumor formation and AKT inhibitor effects. Note that in mock-treated control animals T47D cells (yellow) grow out to form a solid tumor mass (arrow, left), whereas dispersed T47D cells persist in the yolk sac of Akti1/2-treated fish (red circle, right). Transgenic *fli:eGFP* zebrafish are used to allow visualization of interactions with host vessels. Scale bar: 500 μm.

To investigate the relevance of the described AKT/SOX2 molecular axis *in vivo*, we next performed xenotransplantation experiments of human BC cells that were micro-injected into the yolk sac of zebrafish embryos, and quantified tumor formation in dependence of AKT and SOX2. Of note, xenotransplantation into zebrafish has been applied in studies of BC tumorigenicity before [28, 29] and was used here because of its advantages in monitoring *in vivo* tumor induction and drug treatment effects [30]. First, fluorescently labeled control or SOX2-overexpressing T47D cells were administered into the yolk sac of zebrafish embryos at 48 hours post fertilization and tumor formation quantified after 5 days of continuous incubation in the presence of doxycycline (Figure 5A). In line with data from murine studies, SOX2 overexpression enhanced tumor induction also in xenotransplanted fish (Figure 5B). Moreover, treatment with AKT inhibitors was able to fully suppress tumor formation (Figure 5C and 5D) while, in agreement with our *in vitro* findings, induction of *SOX2* expression was able to partially restore tumor formation inspite of AKT inhibition (Figure 5C). Taken together, this series of experiments indicate that AKT regulates BC cell clonogenicity and *in vivo* tumorigenicity via modulation of SOX2 protein levels.

### pAKT and SOX2 proteins physically interact in BC cells

**Figure 6:**
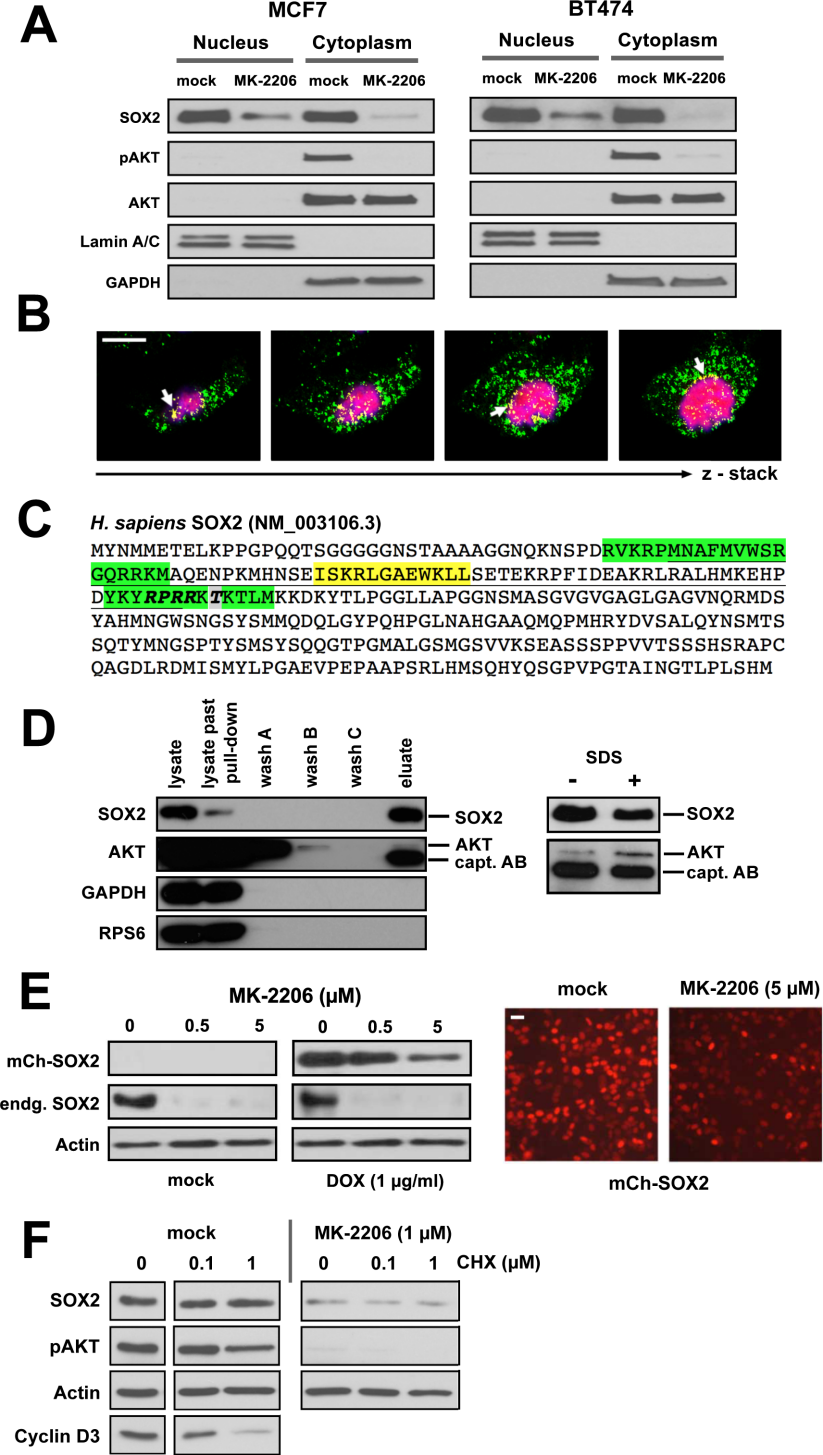
AKT and SOX2 proteins physically interact in BC cells **(A)** Expression and subcellular localization of SOX2, pAKT, and AKT proteins in BC cell lines cultured with or without MK-2206 (5 μM) for 48 hours. Lamin A/C and GAPDH were used as nuclear and cytosolic marker proteins, respectively. **(B)** Confocal laser scanning microscope images demonstrating co-localization of pAKT (green) and SOX2 (red) proteins in MCF7 cells. Shown are consecutive sections of a z-stack recording. Co-localization (yellow) is particularly prominent at the nuclear boundary (arrows). Scale bar: 10 μm. **(C)** Sequence analysis of the human SOX2 protein (NCBI Ref. NM_003106.3). The High Mobility Group (HMG) DNA-binding domain is underlined. Bipartite nuclear localization signal (NLS) in green, nuclear export signal (NES) in yellow. Note an AKT recognition motif (RPRR-X_S/T, bold) and a *bona fide* AKT phosphorylation site Thr116 (grey) within the NLS motif of SOX2. **(D)** Immunoblot analysis documenting co-precipitation of SOX2 and AKT proteins from MCF7 cell lysates and specificity of this interaction over internal controls (GAPDH, RPS6). Note that the detection reagent (peroxidase conjugated anti-rabbit IgG) also stains the capture antibody (rabbit anti-human SOX2). **(E)** MK-2206 treatment inhibits the expression of ectopic SOX2 protein (driven by an exogenous *mCherry-SOX2* fusion construct stably integrated into MCF7 cells), as illustrated by immunoblotting (left) and fluorescence life-microscopy (right). Doxycycline (1 μg/ml) was added for 24 hours to induce the ectopic *mCherry-SOX2* protein, then washed out, and MK-2206 (5 μM) or mock-control added for another 48 hours. Scale bar: 25 μm. **(F)** Induction of a translational arrest by cycloheximide (0.1–1 μM for 48 hours) has only a minor effect on endogenous SOX2 levels. Note that the strong inhibitory effect of MK-2206 on SOX2 protein expression persists in spite cycloheximide-induced translation arrest, indicating direct regulatory effects of pAKT on SOX2 (+/− MK-2206, left vs. right).

Next, we interrogated the molecular basis of the upstream regulatory effect of AKT on SOX2 protein expression. Cell fractionation experiments in different BC lines indicated a nucleo-cytoplasmatic segregation of SOX2 protein at steady-state, and a partial co-fractionation with AKT/pAKT in the cytosol (Figure 6A). Interestingly, treatment with MK-2206 induced a more pronounced relative decline of SOX2 protein in cytosolic as compared to nuclear fractions, suggesting that clearance of SOX2 protein may preferentially occur via the compartment where also pAKT is retained (Figure 6A). This is also supported by confocal laser scanning microscopy, revealing a partial cytoplasmic co-localization of SOX2 and pAKT proteins in particular at the nuclear boundary of BC cells (Figure 6B).

The human SOX2 protein sequence (NM_003106.3) harbors an AKT recognition motif (RPRRX-S/T) and a putative phosphorylation site (Thr116 in human, Thr118 in mouse [31]) near the C-terminal end of its high-mobility-group (HMG) DNA-binding domain (Figure 6C). The AKT recognition motif actually coincides with the nuclear localization signal (NLS) of SOX2 [32], suggesting that AKT may associate with SOX2 to modulate its nuclear entry by phospho-modification. Despite the expected transient nature of such enzyme-substrate relations, we succeeded in confirming a direct physical interaction of SOX2 and AKT proteins by co-immunoprecipitation (Figure 6D). Albeit only a small fraction of total AKT co-enriched with SOX2, the recovery of AKT was shown to be specific over internal controls (GAPDH and RPS6) and increased upon its membrane dissociation with SDS (Figure 6D, right panel). The identity of AKT and SOX2 proteins was further confirmed by peptide fingerprinting (not shown).

To explore the regulation of SOX2 protein by AKT in more detail, the *mCherry-SOX2* protein was lentivirally introduced into BC cell lines and primary cells and its expression induced prior to MK-2206 treatment. As noted for the endogenous protein, AKT inhibition also effectively reduced ectopic SOX2, whose expression was driven from an inducible heterologous promoter (Figure 6E). These data clearly demonstrate that pAKT regulates SOX2 expression by influencing protein turnover. Further supporting the post-translational nature of this effect, cycloheximide treatment efficiently depleted BC cells of proteins with a comparable short half-life (e.g. cyclin D3) but only modestly affected SOX2, indicating that SOX2 protein has a comparably longer half-life in BC (Figure 6F) and a complete inhibition of SOX2 protein could not be achieved as fast through translational repression. We therefore conclude that cells of stalled AKT kinase activity clear SOX2 protein by post-translational mechanisms. Interestingly, whereas this molecular dependence was evident in all BC cell lines and primary samples tested, a comparable tight coupling of SOX2 protein on pAKT activity was not consistently detected in other tumor types (e.g. ovarian and squamous neck carcinoma cell lines), suggesting an involvement of yet unknown tissue-specific factors or even alternative regulatory principles in other cell types (Supplementary Figure 6).

### Proteasomal clearance of cytoplasmic SOX2 upon AKT inhibition

**Figure 7:**
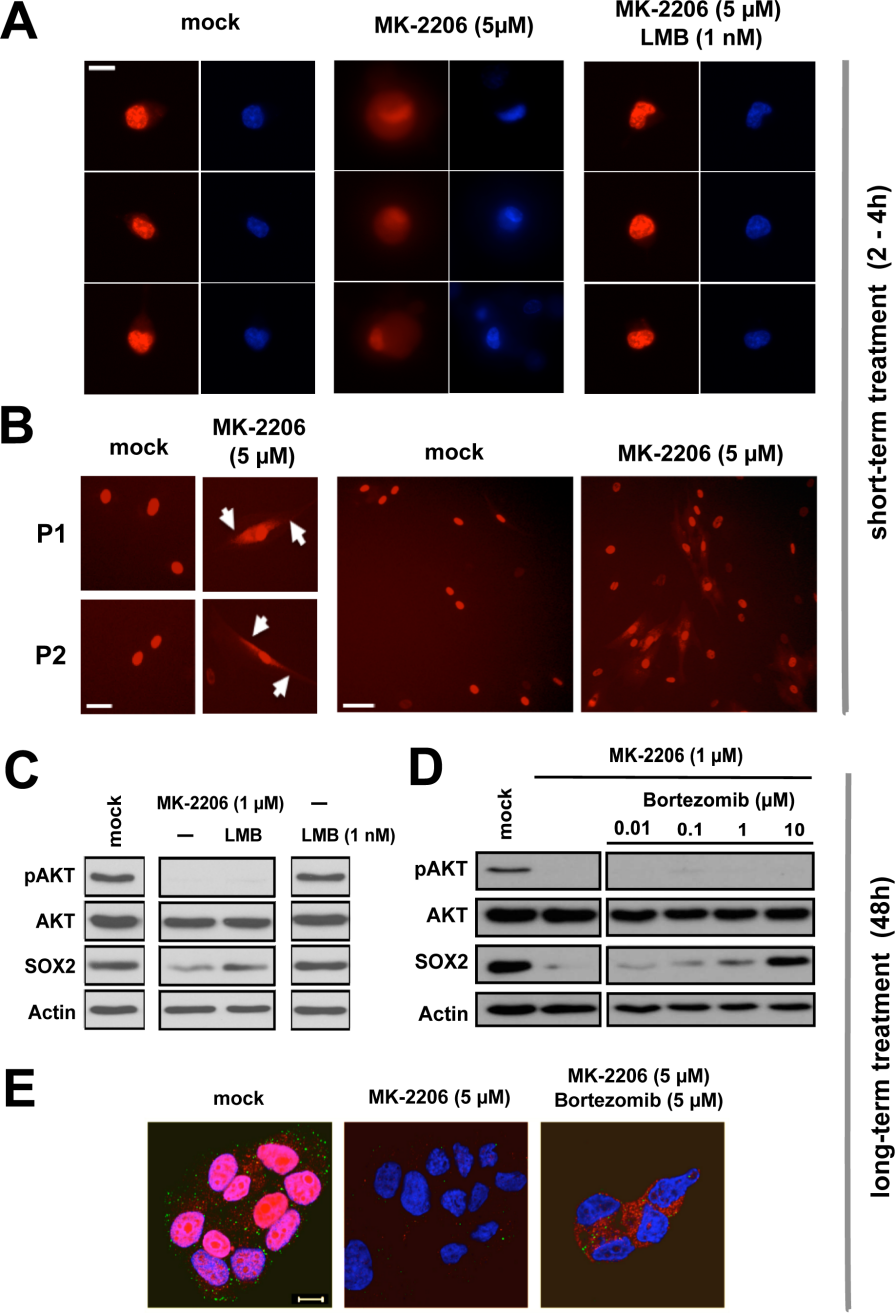
Proteasomal clearance of cytoplasmic SOX2 upon AKT inhibition **(A)** Rapid cytoplasmic accumulation of *mCherry-SOX2* protein signal in BT474 cells treated with 5 μM MK-2206 for 2–4 hours (left to center), and phenotypic restoration upon inhibition of nuclear export with leptomycin B (1 nM, center to right). DNA was stained with Hoechst33342 to indicate nuclei. Scale bar: 10 μm. **(B)** Verification of cytoplasmic *mCherry-SOX2* signal retention in primary patient-derived cells (P1 and P2) treated with MK-2206. Scale bars: 25 μm (left) and 50 μm (right). **(C)** Immunoblot re-confirming depletion of SOX2 protein by MK-2206 (1 μM) and rescue of SOX2 signal in MK-2206 and leptomycin B double-treated cells at 48 hours. **(D)** Immunoblot documenting a dose-dependent rescue of endogenous SOX2 protein in BC cells co-treated with MK-2206 and the proteasome inhibitor bortezomib for 48 hours. **(E)** Corresponding confocal image sections illustrating a depletion of SOX2 protein signal by MK-2206 (left to center) and the restoration of cytoplasmic SOX2 in MK-2206 and bortezomib double-treated MCF7 cells (center to right). Note that in AKT inhibitor-treated cells, bortezomib treatment can rescue SOX2 protein expression but not relocate it to the nucleus. Depicted are cells 48 hours after treatment with the indicated drugs. Red: SOX2; green: pAKT; blue: DAPI. Scale bar: 10 μm.

Live cell imaging visualizing *mCherry-SOX2* protein revealed a bright nuclear signal upon induction with doxycycline (Figure 7A, left), which persisted over several days. In the presence of the AKT inhibitor MK-2206, however, a rapid redistribution of the fluorescent signal from an exclusively nuclear to a nuclear-cytoplasmic signature was observed (Figure 7A and 7B). Cytoplasmic signal retention commenced at about 30 min after the onset of AKT inhibition and became most apparent within 2 to 4 hours. This timing suggested a shifted nucleo-cytoplasmic equilibrium of pre-existing SOX2 protein as predominant cause of signal retention. Indeed, cytoplasmic signal formation was readily abolished and the SOX2 signature effectively retained in the nucleus, when nuclear export was first blocked with leptomycin B and AKT kinase activity stalled with MK-2206 thereafter (Figure 7A, right).

Long-term exposure to MK-2206 for 48 hours instead caused a significant depletion of endogenous SOX2 protein and ectopically expressed *mCherry-SOX2* alike (see Figure 6E for comparison). Interestingly, co-treatment of cells with leptomycin B and MK-2206 allowed for a partial rescue of SOX2 protein even at extended points in time (Figure 7C), suggesting that nuclear retention may have a protective influence on SOX2 protein.

*Vice versa*, we hypothesized that a cytoplasmic accumulation of SOX2 in AKT-inhibited cells promotes its degradation and that proteasomal inhibitors may counteract this effect. To test this assumption, BC cells were treated with MK-2206 with or without the addition of the proteasomal inhibitor bortezomib. Indeed, co-treatment with bortezomib dose-dependently inhibited the MK-2206-induced SOX2 degradation (Figure 7D). These observations were re-confirmed by confocal microscopy of endogenous SOX2 protein that once again documented a disappearance of SOX2 signal upon AKT inhibition, and a restoration of SOX2 protein in BC cells co-treated with MK-2206 and bortezomib (Figure 7E). Notably however, the SOX2 protein was only restored in the cytosol of double-treated cells, indicating that in the presence of AKT kinase inhibition nuclear import of SOX2 was perturbed.

Taken together, our results highlight the importance of the AKT/SOX2 axis for BC clonogenicity and *in vivo* tumorigenicity, and indicate AKT inhibitors as molecules targeting *SOX2*-positive BC (stem) cells via SOX2 protein depletion. Mechanistically, AKT kinase activity promotes SOX2 nuclear entry, thereby influencing its protein turnover (see Supplementary Figure 9 for summary and schematic illustration).

## DISCUSSION

Breast carcinoma is the most common type of cancer and one of the leading causes of cancer death in women worldwide. In spite recent progresses in therapy, BC patients carry a life-long risk of disease recurrence. BC relapse is thought to originate from clonogenic breast CSCs that metastasize, survive anti-tumor therapies and eventually re-initiate disease. Understanding the molecular mechanisms defining breast CSCs may lead to the discovery of molecules effectively targeting this cell population.

The pluripotency-associated protein SOX2 is a key regulator of self-renewal in pluripotent stem cells and was furthermore shown to determine developmental cell fate decisions by interactions with tissue-specific factors [33]. In the adult, SOX2 marks certain stem and progenitor cells important for tissue homeostasis and repair [2, 34, 35]. Recently, an increasing amount of data indicates SOX2 expression in various cancers [3–13]. Here, the SOX2 expression pattern highly depends upon the tissue of origin. In squamous lung carcinoma, for example, SOX2 expression is mostly linked to amplifications at its chromosomal locus 3q26. Consistently, SOX2 is homogenously detected in all tumor cells where it promotes cell growth as a lineage-survival oncogene [4, 10]. In contrast, in breast and ovarian carcinoma SOX2 expression occurs in the absence of *SOX2* gene amplifications and appears enriched in putative CSCs [7, 14]. In line, SOX2 knockdown reduces sphere formation and *in vivo* tumorigenicity in breast as well as ovarian carcinoma cells [11, 25]. Moreover, even breast carcinoma cell lines with inherently low SOX2 levels as observed in 2D cultures (e.g. HS578T or MDA-MB468) are able to activate the gene dynamically when cultured under conditions that promote CSCs (Supplementary Figure 1). This suggests that SOX2 may have an even broader clinical significance in BC than presently anticipated and may regulate also the biology of tumors where no prominent expression is detected in standard screening procedures.

Next to a small set of classical disease-defining genes such as *BRCA-1/2*, the estrogen receptor or *HER2/neu*, the canonical PI3K/AKT/mTOR signaling pathway forms another mutational hotspot in breast cancer [36]. No less than 30–40% of breast cancers contain constitutively active forms of either PI3K or loss-of-function mutations in its upstream suppressor PTEN [26, 37]. This also concerns the particular cell lines investigated here, which either carry a *PI3K-CA* mutation (MCF7, BT474, and T47D) or *PTEN*^−^ alleles (e.g. BT549 and MDA-MB468, see Supplementary Table 1 for comprehensive overview). Underscoring a particular significance of AKT signaling in BC, nuclear stabilization of AKT was recently shown to enhance stem cell-like features in BC cell lines [38]. In line with these results, we observed that, in contrast to conventional cytostatics, treatment with the allosteric pan-AKT kinase inhibitor MK-2206 not only reduced overall BC cell growth, but also suppressed SOX2-expressing putative CSCs and furthermore impaired BC cell clonogenicity and *in vivo* tumorigenicity. Mechanistically, exposure to different PI3K or AKT inhibitors strongly reduced SOX2 protein levels suggesting that PI3K/AKT signaling may regulate breast CSCs via direct modulation of SOX2. In line with this notion and verifying SOX2 as a functional downstream target of AKT in BC, overexpression of SOX2 was able to rescue sphere formation in AKT-inhibited cells, albeit the reduced size of the rescued spheres suggested that other AKT-dependent effects (e.g. induction of cell proliferation) might not be equally restored (see Supplementary Figure 5). Importantly, these data could be confirmed *in vivo* in xenotransplantation experiments where treatment with AKT inhibitors effectively suppressed tumor induction from control, but not from SOX2-overexpressing BC cells.

To explore how pAKT regulates SOX2 expression in BC, an ectopic *mCherry-SOX2* protein was introduced into BC cell lines and primary tumor cells. In the presence of AKT inhibitors, a rapid cytoplasmic accumulation of SOX2 signal along with a relative intensity decline in the nucleus was observed. These effects commenced within minutes after addition of the inhibitor and became most prominent after 2–4 hours of treatment. A putative contribution of *de novo* protein synthesis to this effect cannot be excluded. However, the rapid onset of events and the documented long half-life of SOX2 protein (Figure 6F) emphasize a subcellular redistribution of pre-existing SOX2 protein as the main cause of cytoplasmic signal retention.

At extended incubation times, a successive disappearance of the SOX2 signal in AKT inhibitor-treated cells was noted. The relative decline in SOX2 protein was more pronounced in cytoplasmic than nuclear fractions, suggesting an involvement of cytosolic proteasomal degradation (Figure 6A). Indeed, addition of the proteasomal inhibitor bortezomib to AKT inhibitor-treated cells was able to rescue SOX2 protein expression in the cytosol even at extended incubation times. We therefore conclude that in BC cells AKT modulates SOX2 steady-state levels by counteracting its proteasomal degradation in the cytosol. Underscoring the regulatory role of protein degradation, Wang and co-workers recently defined the ubiquitin-conjugating enzyme Ube2s as a mediator of Sox2 expression in murine ES cells [39].

Mechanistically, our data show that AKT co-localizes and physically interacts with SOX2 and suggest that the nucleo-cytoplasmic distribution of SOX2 is influenced by AKT kinase activity. Supporting this notion, an AKT recognition motif (RPRR-X_T_116_) was identified within the nuclear localization signal of SOX2, emphasizing phosphorylation as a probable means to modulate SOX2 nuclear entry. Of note, an evolutionary conserved phosphorylation site also exists in murine Sox2 and was functionally linked to the reprogramming of murine fibroblasts into induced pluripotent stem (iPS) cells [31] and shown to influence Sox2 protein stability in murine ESCs [40]. Interestingly, a Thr_116_ Ala single-site mutation of this previously reported locus is insufficient to block SOX2 nuclear import in BC cells (Supplementary Figure 7), suggesting an involvement of additional AKT–dependent phosphorylation sites within SOX2, as reinforced by *in vitro* kinase assays (data not shown).

To our knowledge, a correlation between AKT kinase activity and SOX2 nuclear entry has not yet been previously reported. In lack of a decisive phosphorylation site mutant, transport assays involving the nuclear export inhibitor leptomycin B (LMB) were performed to provide experimental evidence of altered SOX2 protein transport in anti-AKT treated cells. Indeed, pre-treatment with LMB prevented MK-2206 induced re-distribution and cytoplasmic retention of *mCherry-SOX2* signal, and at extended incubation times LMB treatment partially restored SOX2 levels, suggesting that the established nuclear retention has a protective effect on SOX2.

In mice, Akt has been suggested to indirectly repress *Sox2* transcription via a regulatory circuit involving FoxO1 [41]. Moreover, AKT was recently reported to modulate *SOX2* transcriptional activity via p27 and a regulatory circuit involving miR-30a in human nasopharyngeal cancers [42]. While these reports jointly underscore a functional correlation of AKT and SOX2, we found no evidence for such molecular interactions in BC cells (Supplementary Figure 8 and data not shown). *Vice versa*, the robust effect of AKT inhibition on SOX2 protein expression that we document here for BC was not consistently observed in other tumor-derived cell types, e.g. in ovarian or squamous head and neck cell carcinoma lines (Supplementary Figure 6). Jointly, these data reinforce the notion that SOX2 regulation occurs in a highly tissue-specific manner and that learning derived from one experimental system may have only limited predictive value for other indications. These observations are in line with the immanent differences in SOX2 expression pattern and function observed in different cancer types (see before), which strongly suggest that also the molecular regulation of SOX2 turnover might likely depend upon the tissue of origin.

The existence of an AKT recognition motif within the human SOX2 amino acid sequence and the experimental confirmation of a direct physical interaction of AKT and SOX2 proteins via co-localization and co-immunoprecipitation strongly suggest an enzyme-substrate relation between the two factors. Moreover, depletion of SOX2 protein and impaired BC clonogenicity required inhibition of AKT kinase itself (as achieved either by MK-2206 or Akti1/2), or of the upstream kinase PI3K by either wortmannin or GCD-0941. Noteworthy, since different inhibitors of AKT or PI3K reduced SOX2 protein in a similar manner, off-target effects are an unlikely explanation for the results presented here. Interestingly, no depletion of SOX2 protein was observed when the mTOR-inhibitor rapamycin was applied. This observation is of particular importance since it indicates relevant differences in drugs designed to target the PI3K/AKT/mTOR-pathway that are currently underway in clinical trials. Moreover, it illustrates that AKT modulates SOX2 protein turnover directly, not indirectly via an mTOR-dependent modulation of protein synthesis, further supporting our previous results.

Finally, we observed that MK-2206 mediated inhibition of pAKT/SOX2 and clonogenicity was sustained throughout serial replating sphere assays, but eventually showed recovery. The transient nature of these inhibitory effects indicates that BC stem/progenitor cells are neither eradicated nor terminally differentiated by the treatment regimen applied here. Whether longer application windows or iterative treatment cycles may indeed induce ultimate cell-fate changes and persistent effects, as recently reported in nasopharyngial carcinoma derived cell lines [42], requires further investigation.

In summary, our investigations uncovered a hitherto unrecognized molecular and functional coupling of AKT and SOX2 proteins that determines tumorigenicity in BC, thus adding a novel perspective onto the promises and limitations of PI3K/AKT/mTOR-inhibitor therapies that are currently under laboratory and clinical investigation in BC.

## MATERIALS AND METHODS

### Cell culture

Cell lines (DSMZ, Braunschweig, Germany) were cultured according to data sheet. Primary BC samples obtained from patients treated at the Women's University Hospital Tuebingen, Germany, were dissociated to single cells as previously described [11] and cultured in RPMI 1640 medium (R8758, Sigma, St-Louis, MO, USA) supplemented with 15% heat-inactivated FCS (#10500, Gibco, Life Technologies, Grand Island, NY, USA) and 1% v/v Pen/Strep (P4333, Sigma). The study was approved by the Ethics Committee of the University of Tuebingen, Germany. MK-2206, wortmannin, rapamycin, bortezomib (all by Selleckchem, Houston, TX, USA) or Akti1/2, leptomycin B, cycloheximide, and doxycycline (all by Sigma) were resolved or diluted according to data sheet and applied as indicated.

### Sphere assay and 3D-culture

Sphere assays were conducted in MEBM medium (CC-3151, Lonza, Basel, Switzerland) supplemented with 4 μg/mL heparin (Ratiopharm, Ulm, Germany), 1x hydrocortisone (CC-4031G, Lonza), 1x insulin (CC-4021G, Lonza), 2% B-27 (#17504, Gibco, Life Technologies), 20 ng/ml EGF (E9644, Sigma), 20 ng/ml basic FGF (#100-18B, PeproTech, Rocky Hill, NJ, USA), and antibiotics. Unless indicated differently, 1250 cells were seeded into 300 μl medium and propagated in 24-well ultra-low attachment plates (#3473, Corning, NY, USA) at 37°C and 5% CO_2_. Sphere numbers were quantified at assay day 5 (i.e. after 120 hours of continuous incubation). Live single cells from trypsinized spheres were used for replating assays. For 3D-cultures, 5 × 10^5^ cells were transferred to 10 ml sphere medium and propagated in 25-cm^2^ flasks (#3815, Corning) for 5 days, passaged by trypsinization, and analyzed after another 5 days of sphere cultivation.

### Genetic modifications

For genetic manipulation of cells, lentiviral particles encoding *SOX2* shRNAs and GFP [11] or the SRR1-dsRED reporter and a puromycine resistance cassette [24] were produced and used for cell transduction as previously described. In particular, the following sequences were used to generate *SOX2* shRNAs: sh1_fwd: 5′-GATCCCCCAAGGAGAGGCTTCTTGCTGAATTTTTCAAGAGAAAATTCAGCAAGAAGCCTCTCCTTGTTTTTGGAAA-3′; sh1_rev: 5′-AGCTTTTCCAAAAACAAGGAGAGGCTTCTTGCTGAATTTTCTCTTGAAAAATTCAGCAAGAAGCCTCTCCTTGGGG-3′; sh2_fwd:5′-GATCCCCCGAGATAAACATGGCAATCAATTCAAGAGATTGATTGCCATGTTTATCTCGTTTTTGGAAA-3′; sh2_rev: 5′-AGCTTTTCCAAAAACGAGATAAACATGGCAATCAATCTCTTGAATTGATTGCCATGTTTATCTCGGGG-3′. Human *SOX2* cDNA fused N-terminally to *mCherry* was cloned into a Tet_on_ lentiviral gene induction system (Clontech, Mountain View, CA, USA) driven by doxycycline (D9891, Sigma). Phosphorylation-deficient *SOX2* T116A was obtained by site-directed mutagenesis. A myristoylated AKT construct (Addgene, Cambridge, MA, USA) transiently introduced via co-transfection with lipofectamine 2000 (Invitrogen, Life Technologies) was used to overexpress AKT. Efficiently transduced cells were positively selected by antibiotic resistance and FACS.

### Real-time PCR

Total RNA was isolated with the RNeasy Mini Kit (Qiagen, Valencia, CA, USA) and cDNA synthesized using a high capacity cDNA reverse transcription kit (Applied Biosystems, Life Technologies). qRT-PCR was performed on an ABI 7500 Light Cycler (Applied Biosystems, Life Technologies) using the FastStart Universal SYBR Green Master mix (Roche, Basel, Switzerland) and the following primer sets for detection of indicated marker genes: *SOX2* (fwd, 5′-AAGACGCTCATGAAGAAGGATAA-3′; rev, 5′-ACTGTCCATGCGCTGGTT-3′), *GAPDH* (fwd, 5′-CTGACTTCAACAGCGACACC-3′; rev, 5′-TAGCCAAATT CGTTGTCATACC-3′), *beta-Actin* (fwd, 5′-AGTCCTGTGGCATCCACGAAAC T-3′; rev, 5′-CACTGTGTTGGCGTACAG GTCTT-3′), and *AKT1* (QuantiTect primer set QT00085379, Qiagen). Expression levels relative to *GAPDH* were calculated using the ΔΔ*C*_T_ method.

### Immunoblotting

Cells were disrupted in 1x Lysis Buffer (#9803, Cell Signaling, Danvers, MA, USA) supplemented with Protease/Phosphatase Inhibitor Cocktail (#78442, Thermo Fisher Scientific, Waltham, MA, USA). Total protein was precipitated and denatured in Laemmli buffer, separated over 12% bis-acrylamide (#161–0148, BioRad, Hercules, CA, USA) gels by Disc-SDS-PAGE, and transferred onto PVDF membrane (#10600021, Amersham, GE Healthcare Life Sciences, Chalfont St. Giles, UK) in a semi-dry blotting apparatus (Trans-Blot Turbo, BioRad). Membranes were blocked with 10% w/v nonfat dry milk (#9999S, Cell Signaling) diluted in TBS 0.1% Tween-20 (p1379, Sigma). Proteins were stained with the following primary antibodies (all by Cell Signaling): anti-SOX2 [either #3579S (rabbit) or #4900S (mouse)], anti-pan AKT (#4691S), anti-pAKT (i.e. pSer473, #4060S), anti-pRPS6 (#4858), anti-Actin (#3700S), anti-lamin A/C (#4777S), anti-GAPDH (#5174P) and detected either by ECL reaction or phospho-imaging. Cell fractionation analyses were performed with a NE-PER Nuclear and Cytoplasmic Extraction kit (Thermo Fisher Scientific) according to the manufacturer's instructions.

### Immunoprecipitation

MCF7 cells (100 mg wet pellet weight) were disrupted in Tris/HCl-based Cell Lysis Buffer (#9803, Cell Signaling) supplemented with 1x Protease/Phosphatase Inhibitor Cocktail (#78442, Thermo Fisher Scientific) and incubated with 1 μl of capture antibody (rabbit anti-human SOX2, #3579S, Cell Signaling) for 16 hours at 4°C. Bait-antibody complexes were precipitated with 50 μl (50% slurry bead volume) equilibrated Protein A-Agarose Fast Flow Beads (#92529, Miltenyi Biotech, Bergisch Gladbach, Germany) for 1 hour at 4°C. Bead-protein complexes were sedimented (5 min, 1200 rpm, 4°C) and iteratively (3x) washed with 1 ml cold buffer to resolve non-specifically interactions. Cleared bead-antibody-bait complexes were re-suspended in 100 μl Laemmli buffer, denatured at 95°C for 5 min, and analyzed by immunoblotting.

### Microscopy

For life cell microscopy, expression of the *mCherry-SOX2* protein was induced with 1 μg/ml of doxycycline for 24 hours and the medium exchanged preceeding anti-AKT treatment. Images were either recorded at 2–4 hours to document cytoplasmic retention of SOX2 (short-term treatment), or after 48 hours to document SOX2 protein decay in dependence of AKT (long-term treatment). For immunofluorescence, cells fixed in 4% PFA were permeabilized with 0.1% Triton, stained with antibodies and analyzed. Life cell imaging was performed on IX-50 and IX-81 microscopes (U-RFL-T laser, Olympus, Tokyo, Japan) and confocal images recorded with a LSM 710 microscope (Carl Zeiss, Oberkochen, Germany). Data were processed in ImageJ software (http://imagej.nih.gov/ij) and co-localization analyzed with Zeiss Zen software.

### Zebrafish xenografts

Animal experiments and zebrafish husbandry were approved by the “Kantonales Veterinaeramt Basel-Stadt”. T47D cells were labeled with the fluorescent CellTracker™ CM-DiI (Life Technologies), a lipophilic fluorescent tracking dye, according to the manufacturer's instructions. Tg (*flk1:eGFP*) zebrafish were maintained, collected, grown and staged in E3 medium at 28.5°C according to standard protocols [43]. For xenotransplantation experiments, zebrafish embryos were anesthetized in 0.4% tricaine (Sigma) at 48 hours post fertilization (hpf) and 75 T47D human BC cells micro-injected into the vessel-free area of the yolk sac. Embryos were incubated for 1 hour at 28.5–29°C for recovery and cell transfer verified by fluorescence microscopy. Fish harboring red cells were incubated at 35°C essentially as described before [30, 44] and the water supplemented with 1 μM Akti1/2 (Sigma) or DMSO at day 0 and day 2.5 post transplantation. On assay day 5, embryos were screened microscopically for tumor cell engraftment using a Zeiss LSM 710 confocal microscope and the number of tumor-bearing fish quantified. For rescue experiments, expression of a *mCherry-SOX2* fusion protein was induced with 1 μg/ml of doxycycline (Sigma) for 24 hours and protein formation verified by fluorescence microscopy prior to transplantation.

### Statistics

Unless otherwise indicated, data from ≥ 3 independent biological triplicates was analyzed using the student's *T*-Test *p* ≤ 0.05 (*), *p* ≤ 0.001 (**), *p* ≤ 0.0001 (***). Primary cells were analyzed in technical triplicates. Error bars indicate standard deviations (SD).

## SUPPLEMENTARY MATERIALS FIGURES AND TABLES


