# Supplementary material for: Molecular and functional interactions between AKT and SOX2 in breast carcinoma
Source: Oncotarget. 2015 Oct 20;6(41):43540–56. doi: 10.18632/oncotarget.6183 (PMC4791249; doi:10.18632/oncotarget.6183)
Supplement: Supplementary file 1 [file oncotarget-06-43540-s001.pdf]

Molecular and functional interactions between AKT and SOX2 in breast carcinoma

Supplementary Materials

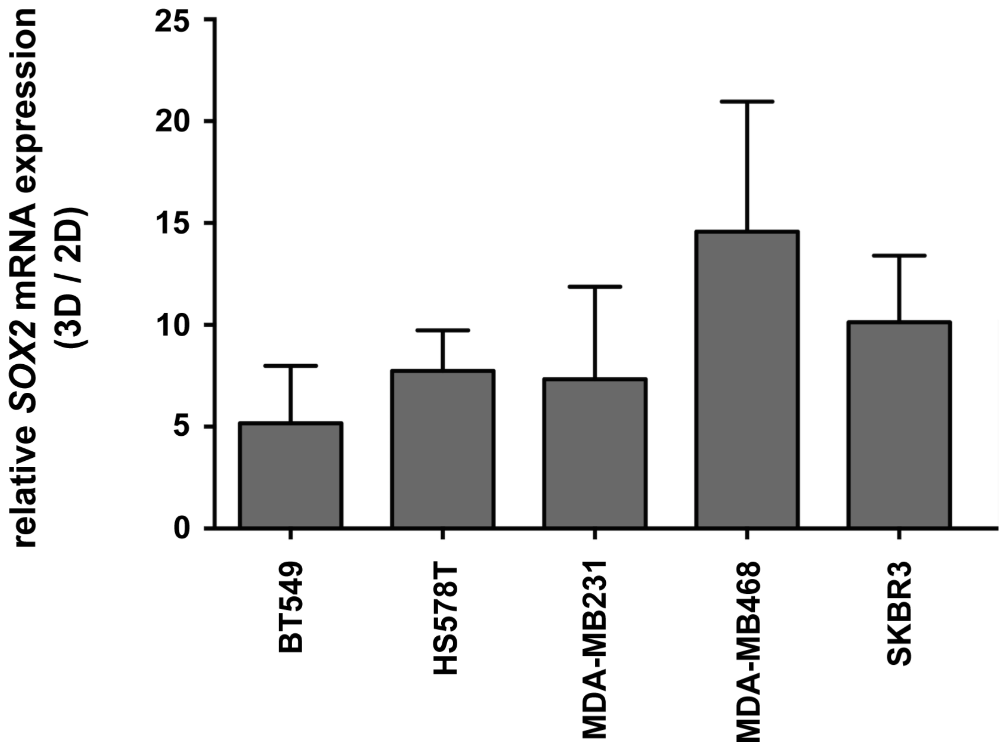

Supplementary Figure S1: Induction of *SOX2* gene expression in *SOX2*-negative/low BC cell lines grown under 3D- versus 2D-culture conditions. Real-time PCR-based quantification of ***SOX2*** mRNA in the indicated BC cell lines grown under CSC-enriching 3D-conditions or standard 2D-conditions. Shown are fold changes in relative *SOX2* expression levels in 3D versus 2D cultures normalized to *GAPDH* expression, as deduced from three independent experiments.

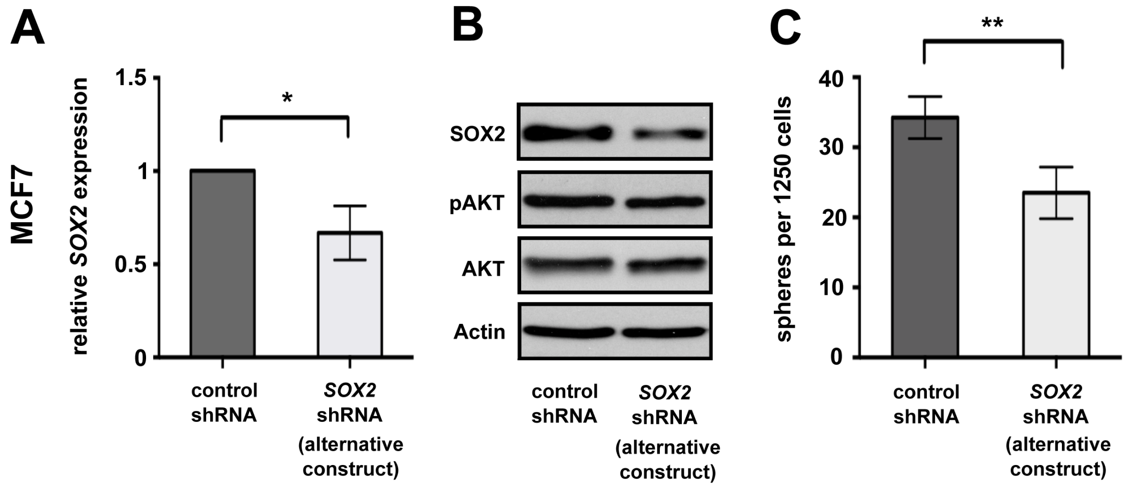

Supplementary Figure S2: Verification of *SOX2* knockdown phenotypes using an alternative shRNA. (**A**) Reduced *SOX2* mRNA and (**B**) protein expression, and (**C**) impaired sphere formation in MCF7 cells transduced with an alternative *SOX2* shRNA vs. control lentiviral particles. Note essentially unaltered (p)AKT levels in cells with impaired *SOX2* expression (B).

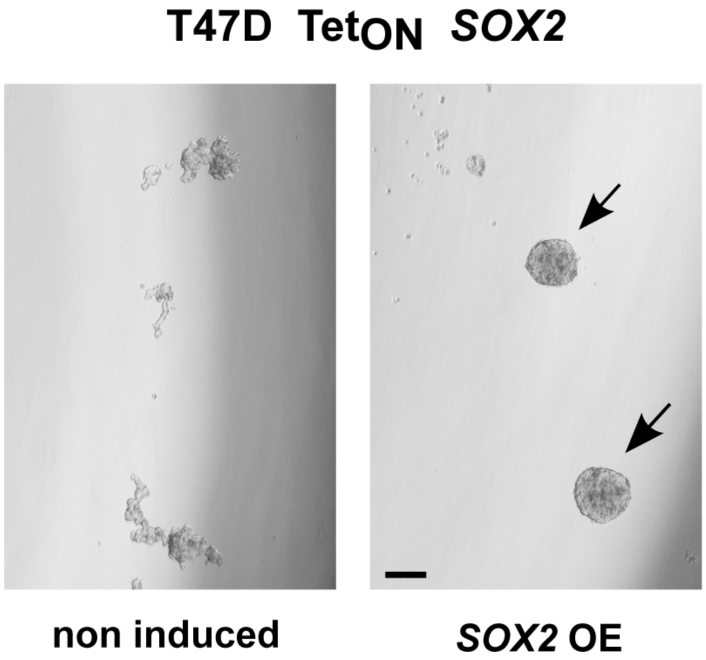

Supplementary Figure S3: *SOX2* expression induces sphere formation from T47D cells. Overview images documenting the supportive role of *SOX2* expression on sphere formation from T47D cells. Shown are representative pictures of mock-treated (non induced, left) and *SOX2*-overexpressing T47D cells (1 µg/ml doxycycline, right) after 5 days of cultivation in sphere assay medium. Scale bar: 100 µm.

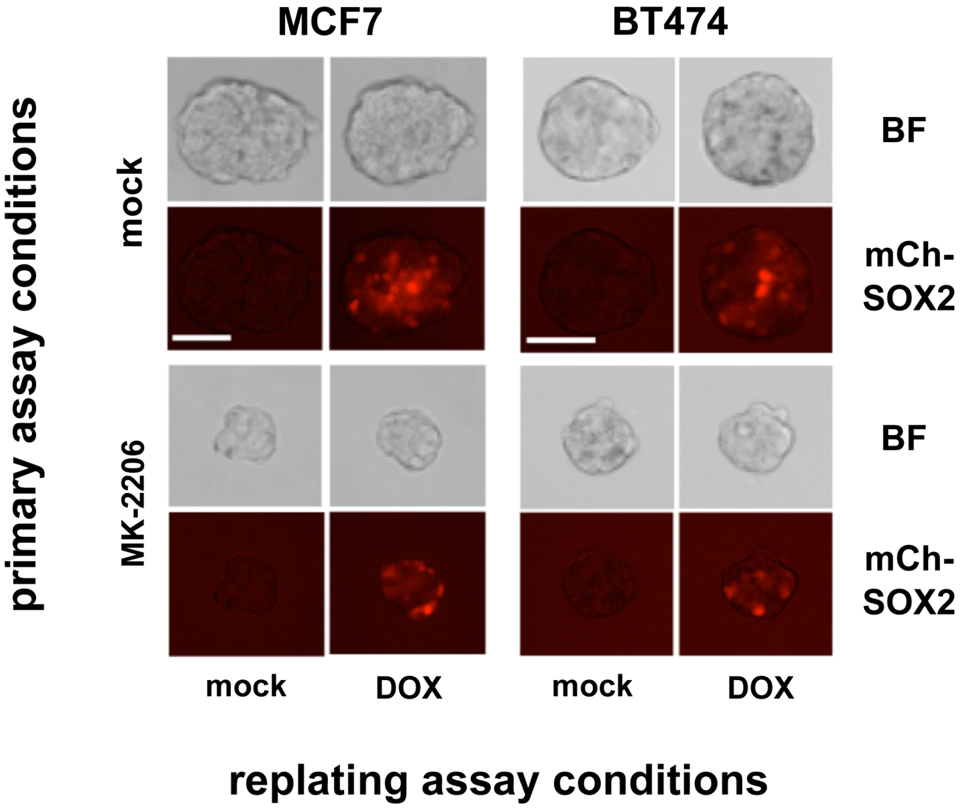

Supplementary Figure S4: Verification of inducible mCherry-SOX2 protein expression in replated spheres. Shown are representative single sphere images of MCF7 and BT474 cells derived from replated spheres (1st replating assay), documenting effective SOX2 protein induction in response to addition of 1 µg/ml doxycycline (DOX) to 3D cultures. Samples were treated with 5 µM MK-2206 or mock control in the first sphere assay, and subsequently re-seeded into mock medium with or without DOX. Note a reduction in sphere size upon MK-2206 treatment (bright field, BF, top panels), and a robust induction of the fusion protein by doxycycline as verified by fluorescent microscopy (mCherry-SOX2, bottom panels). Scale bars: 100 µm.

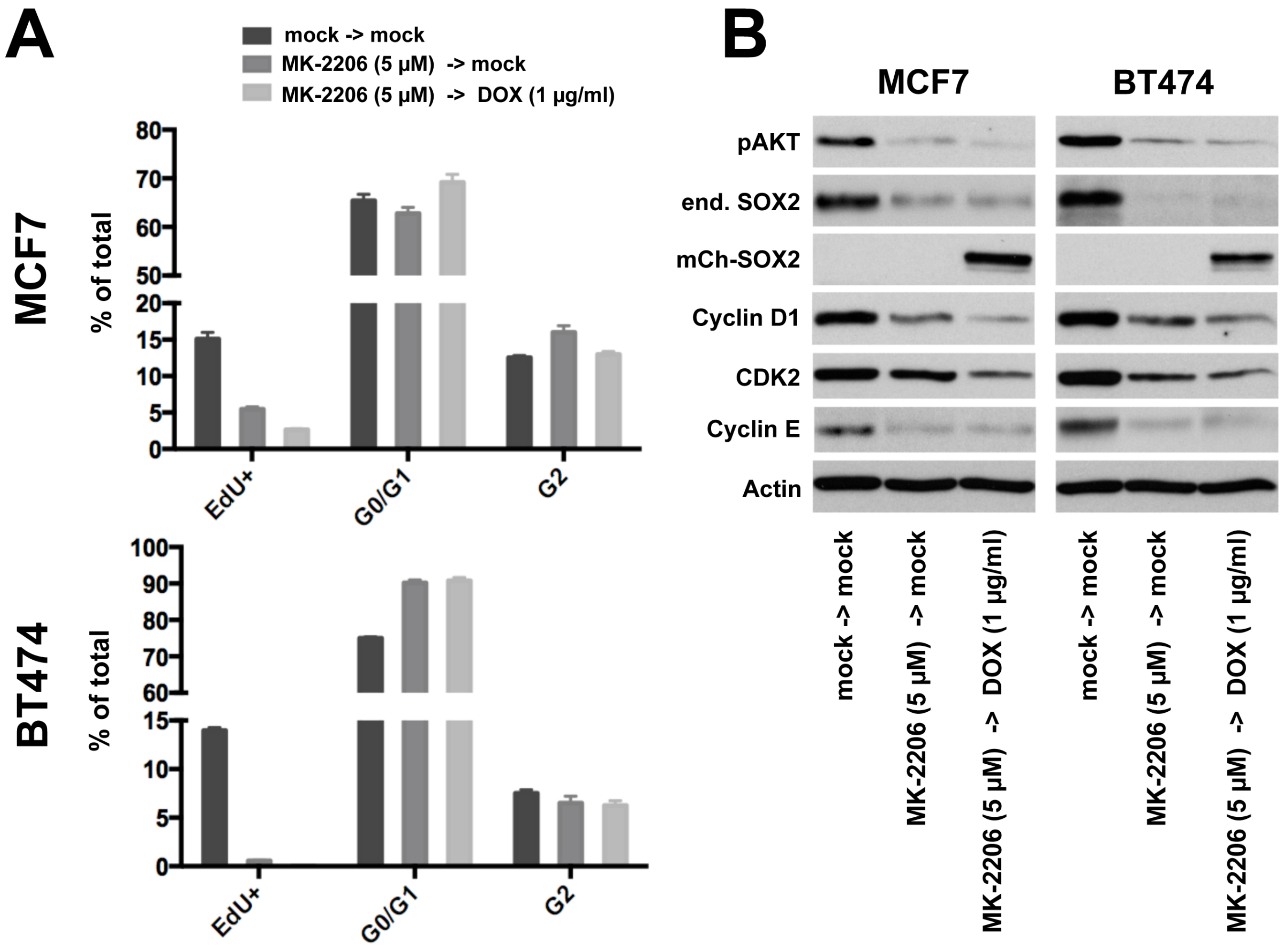

Supplementary Figure S5: Ectopic *SOX2* induction fails to restore proliferation defects in AKT-inhibited cells. (**A**) Cell-cycle analysis of the indicated BC cell lines grown for 2.5 days with or without the AKT inhibitor MK-2206 (5 µM) followed by further 2.5 days of incubation in fresh medium in the absence or presence of doxycycline (1 µg/ml) to induce expression of mCherry-SOX2. Cells were labeled with 5-ethynyl-2′-deoxyuridine (EdU) for 2 hours and stained with DAPI prior to flow cytometry. Note that inhibition of AKT imposes a proliferation defect on cells that is not restored by SOX2. (**B**) Corresponding immunoblot analyses documenting perturbed expression of key cell cycle regulators (i.e. cyclin D1, cyclin E, and CDK2) in AKT-inhibited cells irrespective of SOX2 expression.

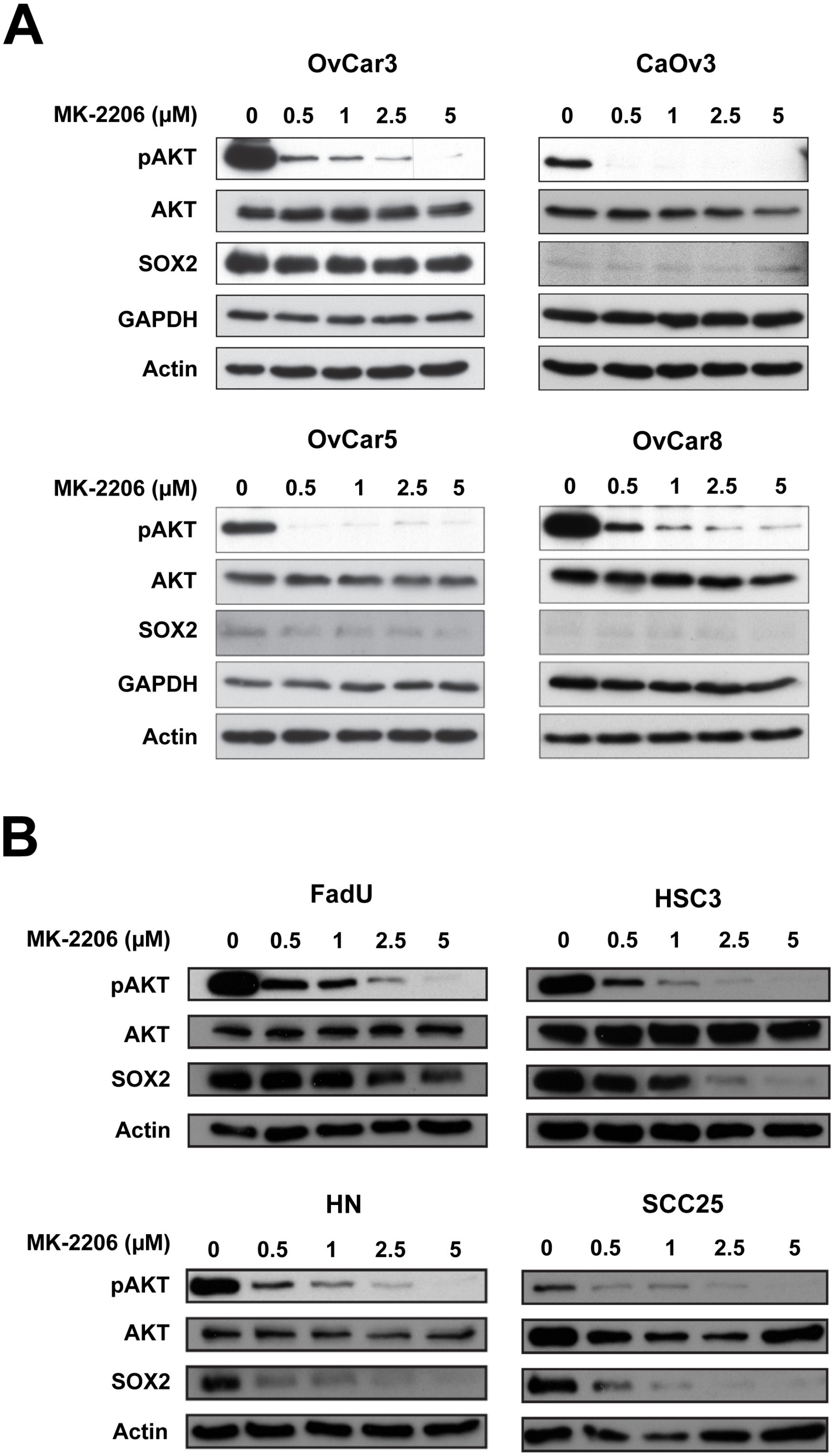

Supplementary Figure S6: Variable effects of AKT inhibition on SOX2 protein levels in other tumor entities. Side-by-side analysis of pAKT, AKT, and SOX2 protein levels upon 48 hours treatment with different doses of MK-2206 in four ovarian (OvCar3, CaOv3, OvCar5, and OvCar8, A) and four squamous head and neck cell carcinoma cell lines (FadU, HSC3, HN, and SCC25, B). Anti-GAPDH and/or anti-actin staining are shown for loading control.

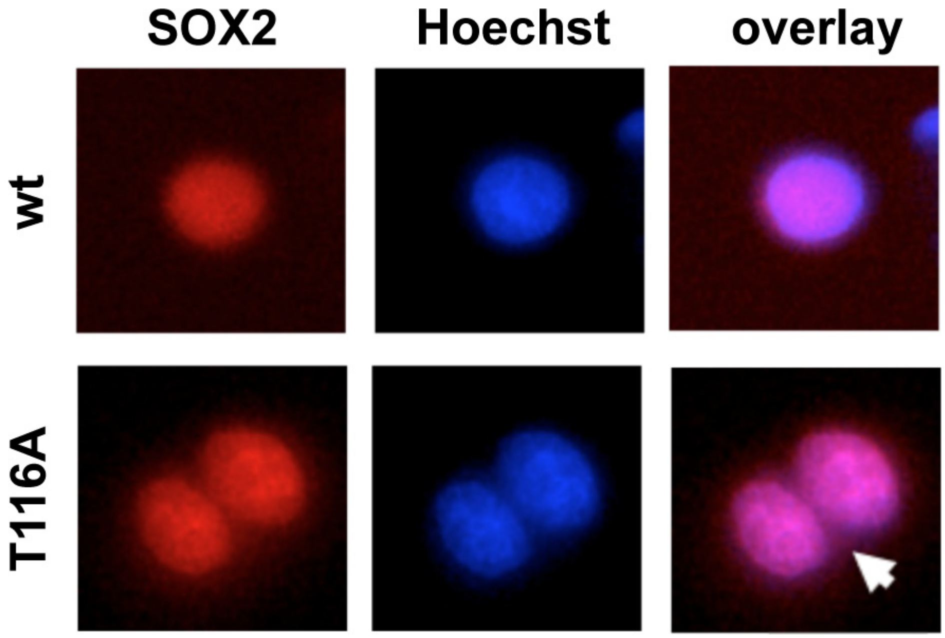

Supplementary Figure S7: Subcellular localization of a mCherry-SOX2 (T116A) phosphorylation site mutant. Live cell microscopic investigation of the steady-state subcellular localization of inducible mCherry-SOX2 constructs (red) in wild-type (wt) or T116A mutant MCF7 cells 24 hours after addition of doxycycline (1 µg/ml). Nuclei were stained with DAPI (blue). Note a bright nuclear staining indicative of persisting nuclear import also of the mutant SOX2 protein arrow. Scale bar: 10 µm.

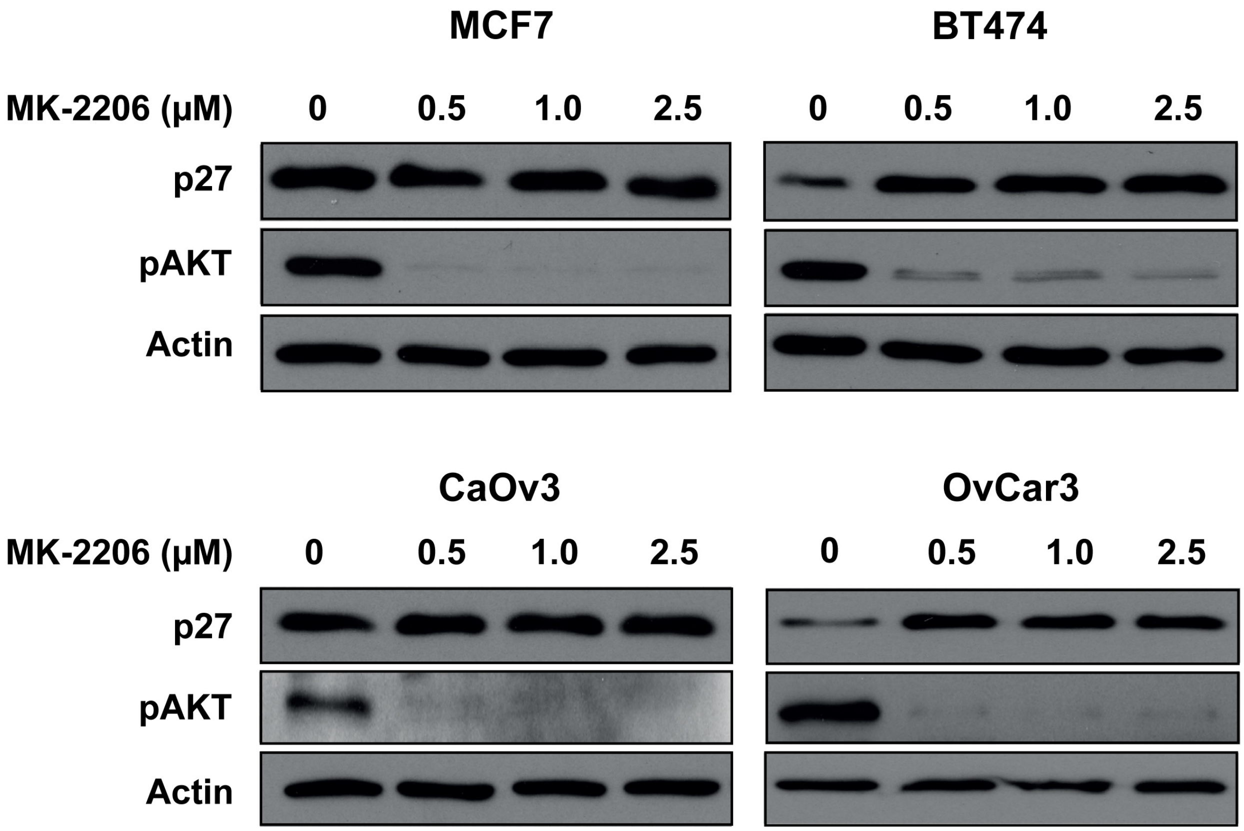

Supplementary Figure S8: No consistent correlation of p27 and pAKT expression in breast and ovarian carcinoma cell lines. Side-by-side analysis of p27 and pAKT protein levels 48 hours upon treatment with the indicated doses of MK-2206 in two breast (MCF7, BT474; top) and two ovarian carcinoma cell lines (CaOv3, OvCar3; bottom). Note that p27 expression is inconsistently affected by AKT kinase inhibition in these tumor cell lines. Anti-actin staining is shown for reference.

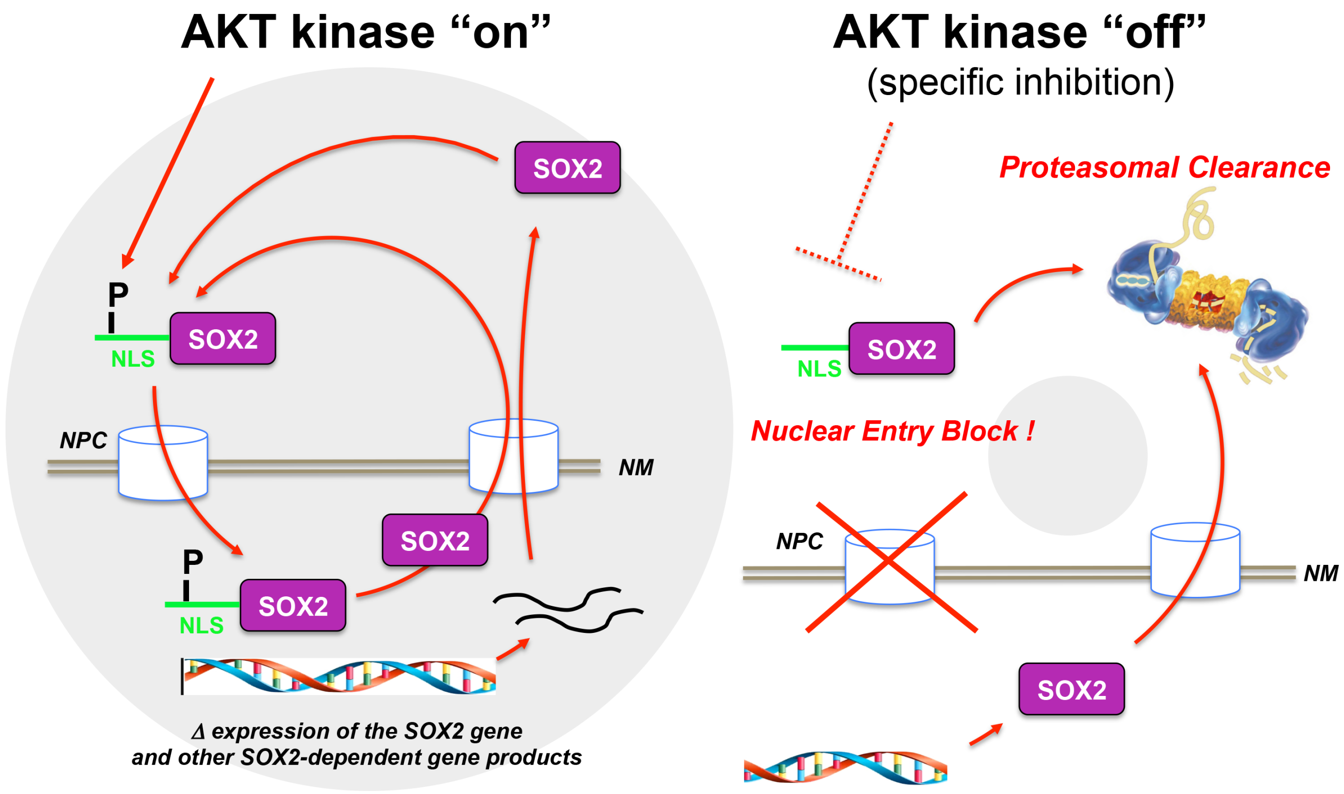

Supplementary Figure S9: AKT kinase determines BC clonogenicity via regulation of SOX2 nuclear entry and protein turnover. AKT kinase activity (left) promotes SOX2 nuclear entry likely involving phosphor-modification of the SOX2 nuclear import signal (NLS). At steady-state, an equilibrium between nuclear entry and export of SOX2 protein is established that involves auto-regulatory mechanisms. AKT kinase inhibition (right) induces a nuclear entry block of SOX2, leading to (i) a rapid cytoplasmic retention of pre-formed SOX2 protein and (ii) to a gradual proteasomal clearance of SOX2 in the cytosol. A functional correlation between SOX2 protein expression and BC cell clonogenicity, as deduced from tumor formation assays *in vitro* and *in vivo*, is illustrated by grey lobes. NPC, nuclear pore complex; NM, nuclear membrane.

Supplementary Table 1: Human breast cancer cell lines used in this investigation. Index list of the human breast cancer cell lines investigated with reference to parental tumor source, classification, and standard gene markers [1–3]

Cell Line	Primary Tumor	Classification	*ER* +/−	*HER2* +/−	*PR* +/−	*PTEN*[*loss*]	*PI3KCA*	*Mutant TP53*
BT474	Invasive ductal carcinoma	Luminal B	+	+	+	−	+	+
BT549	Invasive ductal carcinoma	Claudin-low	−	−	−	+		+
HS578T	Carcinosarcoma	Claudin-low	−	−	−			+
MCF-7	Invasive ductal carcinoma	Luminal A	+	−	+	−	+	−
MDA-MB231	Invasive ductal carcinoma	Claudin-low	−	−	−	−	−	+
MDA-MB468	Adenocarcinoma	Basal	−	−	−	+	−	+
SKBR3	Invasive ductal carcinoma	HER2	−	+	−	−	−	+
T47D	Invasive ductal carcinoma	Luminal A	+	−	+	−	+	+

Supplementary Table 2: Histopathological characteristics of the investigated primary breast carcinoma samples P1 and P2

Patient	Histology	subtype	ER status	PR status	HER 2 status	Tumor size	Grading	Nodal Status
P1	Labular	Luminal	+	+	−	pT2	II	neg
P2	Ductulolobular	HER2	+	+	+	pT2	II	neg

REFERENCES

1. Holliday DL, Speirs V. Choosing the right cell line for breast cancer research. Breast Cancer Res. 2011; 13:215.
2. Lacroix M, Leclercq G. Relevance of breast cancer cell lines as models for breast tumours: an update. Breast Cancer Res Treat. 2004; 83:249–289.
3. Sangai T, Akcakanat A, Chen H, Tarco E, Wu Y, Do KA et al. Biomarkers of response to Akt inhibitor MK-2206 in breast cancer. Clin. Cancer Res. 2012; 18:5816–5828.
